# CARE PLAN INVENTORY AND ASSESSMENT—A MEANS TO LEARN MORE ABOUT CMS

**DOI:** 10.1093/geroni/igad104.0024

**Published:** 2023-12-21

**Authors:** Maria Carney

**Affiliations:** Northwell Health, Manhasset, New York, United States

## Abstract

The Health and Aging Policy Fellowship (HAPF) provides an orientation on the federal government organization and an introduction to aging policy foundations and agencies. The goal of this orientation is to lead to a growth of a professional network as well as a policy placement opportunity. I will explain the process for choosing a HAPF placement with the Centers for Medicare and Medicaid Services (CMS) and Agency for Health Research and Quality (AHRQ). Using my HAPF experience as an example of how my interest in Age-Friendly Health System work at a large health system led to my placement and personal growth. I will discuss my reasoning in taking part in the Fellowship and the creation of CMS Care Plan Inventory and Assessment. Achieving goal-concordant care is the bedrock of Age-Friendly care. Creating care plans is a means for teams and insurance providers use to create goal concordant care. Measuring goal concordant care is a challenge, but development of care plans would be a potential start. CMS utilizes care planning in programs and in billing codes. My Health and Aging Policy Fellowship placement assessed how care planning is defined and what are the elements in CMS programs and billing codes and if these are aligned with care plan research from AHRQ. My experience, current interests and project development will be the example of how HAPF provided me with a better understanding of CMS programs and how billing code creation affects clinician behaviors and , in turn, patients.

